# A comparison between the effects of simulation of basic CPR training and workshops on firefighters’ knowledge and skills: experimental study

**DOI:** 10.1186/s12909-024-05165-z

**Published:** 2024-02-23

**Authors:** Amir Faghihi, Zeinab Naderi, Mohammad Mehdi Keshtkar, Leila Nikrouz, Mostafa Bijani

**Affiliations:** 1 Department of Medical Surgical Nursing, School of Nursing, Fasa University of Medical Sciences, Fasa, Iran; 2 Department of Medical Surgical Nursing, Sirjan School of Medical Sciences, Sirjan, Iran; 3 Student Research Committee, Fasa University of Medical Sciences, Fasa, Iran

**Keywords:** Basic life support, Firefighters, Cardiopulmonary resuscitation, Simulation, Education

## Abstract

**Background:**

One of the most common causes of death worldwide is cardiopulmonary arrest. Firefighters are among the first responders at the scenes of accidents and can, therefore, play a key part in performing basic cardiopulmonary resuscitation (CPR) for victims who need it. The present study was conducted to compare the effects of simulation training against workshops on the CPR knowledge and skills of firefighters in the south of Iran.

**Methods:**

This experimental (Interventional) study was conducted on 60 firefighters of south of Fars province, Iran. The study was undertaken from March to July 2023. Through random allocation, the participants were divided into two groups: simulation-based training (30 members) and traditional workshop training (30 members). The participants’ CPR knowledge and practical skills were measured before, immediately after, and three months after intervention.

**Results:**

The findings of the study revealed a statistically significant difference between the pretest and posttest CPR knowledge and skill mean scores of the simulation groups as compared to the workshop group (*p* < 0.001). As measured three months after the intervention, the firefighters’ knowledge and skill mean scores were still significantly different from their pretest mean scores (*p* < 0.001); however, they had declined, which can be attributed to the fact that the study population did not frequently exercise CPR.

**Conclusion:**

Based on the findings of the study, even though both methods of education were effective on enhancing the firefighters’ CPR knowledge and skill, simulation training had a far greater impact than training in workshops. In view of the decline in the participants’ knowledge and skill scores over time, it is recommended that short simulation training courses on CPR should be repeated on a regular basis.

## Introduction

Cardiopulmonary arrest continues to be a prevalent sudden cause of death in many countries [1]. In recent decades, the rate of deaths due to cardiovascular diseases has declined in high-income countries, with 50% of mortalities and 80% of the global burden from cardiovascular diseases occurring in low- and middle-income countries, especially in east Mediterranean countries [2]. In the Middle East, 46% of the total deaths and 20% of the burden caused by cardiovascular diseases belong to Iran, which ranks first in the region in that regard [3].

The survival rate and neurological consequences of cardiopulmonary resuscitation (CPR) are closely linked to the quality of CPR and timeliness of other medical services [4]. CPR plays a crucial part in preventing 25% of fatalities, especially deaths outside hospitals [5]. As correct and timely CPR can significantly lower the rate of death due to cardiac arrest, performing CPR is a vital skill an individual should acquire [6]. According to a review of literature, life-saving techniques, including CPR, in pre-hospital settings is more challenging than inside hospitals and several studies report that the quality of pre-hospital CPR is generally low [7].

The continuing advances in medical sciences, alongside the emergence of new teaching and learning theories and approaches, have created new responsibilities for healthcare administrates and educators, with one of the most important ones being selecting the best method of learning [8]. The patients’ characteristics as well as the personal and professional characteristics of the members and management of resuscitation teams are known as the main causes of low success rate of CPR in Iran. Thus, there is an urgent need for standard retraining programs designed to update the personnel’s knowledge and skills as well as formation of competent and coordinated resuscitation teams. Assessment and enhancement of individuals’ CPR knowledge and skills can contribute to a higher success rate of CPR and reduction in fatalities plus complications related to CPR [9, 10].

In various incidents, natural or otherwise, firefighters are among the first responders who are dispatched to the scene and play a key part in saving lives by performing basic life support. These professionals are responsible for performing important activities in pre-hospital care, including providing prompt and appropriate care, stabilizing the patient’s vital signs, reducing morbidity and mortality, and transporting the patient to a medical facility as fast as possible [11]. Studies show that, despite being trained in basic life support (BLS) techniques, many firefighters still cannot perform the procedures correctly, with one of the main reasons being trainers’ use of traditional learning approaches [12, 13]. Adopting the best learning approach for improving the learners’ knowledge and skill has always been a challenge in CPR training courses [14].

Simulation, a technique for creating an experience without going through an actual event, provides opportunities which are not often available in clinical environments and allows for learning in a safe and stress-free environment [15]. Over the past two decades, learning via simulation has gained prominence in clinical courses: today, most of the clinical training of target groups is conducted via this approach. Use of computer-assisted educational programs, advanced manikins, as well as videos and slides is part of simulation training programs and considered to be one of the most effective ways to achieving lasting acquisition of knowledge and skills by care givers [16]. In addition, video simulations of CPR scenarios of patients with cardiac arrest in hospitals can contribute to paramedics’ competence, even if the pictures are of poor quality [17].

## Background in IRAN

In Iran, pre-hospital emergency personnel are not permitted to enter the hot zone. The responsibility of rescuing patients at the scene and transporting the injured to the cold zone falls on firefighting personnel. Unfortunately, the training program for firefighting operational personnel does not include any training related to basic cardiopulmonary resuscitation. Therefore, it is vital to teach firefighters the basic concepts of medical emergencies, especially basic cardiopulmonary resuscitation.

Several quantitative studies have addressed the effects of modern methods of CPR training on firefighters’ skills. In view of the critical role of these professionals’ CPR knowledge and skills in pre-hospital missions, the present study was conducted to compare the effects of simulation training against workshops on the CPR knowledge and skills of firefighters in the province of Fars in southern Iran.

## Objective

Comparing the effects of simulation basic CPR training and workshops on firefighters’ knowledge and skills.

### Research questions

Will there be a difference between the pretest and posttest CPR knowledge mean scores of firefighters who are educated via simulation and firefighters who are educated via workshops?

Will there be a difference between the pretest and posttest CPR skills mean scores of firefighters who are educated via simulation and firefighters who are educated via workshops?

## Materials and methods

### Study design

The present study is experimental (Interventional) which was conducted in the south of Fars province, Iran. The study was conducted from March to July 2023. The sample size was calculated using the results of a study by Khademian et al. [18]. The post-intervention means and standard deviations of CPR knowledge in the two groups were 4.82 ± 1.92 and 3.46 ± 1.64, respectively. Considering α = 0.05, 1-β = 0.9, S1 = 1.92, S2 = 1.64, μ1 = 4.82, and μ2 = 3.46. Accordingly, 30 participants were calculated to be needed for each group, with the number raised to 40 to enhance the power and allow for the possibility of loss to follow-up.

$$n=\frac{{\left({Z}_{1-\frac{\alpha }{2}}+{z}_{1-\beta }\right)}^{2}\left({s}_{1}^{2}+{s}_{2}^{2}\right)}{{d}^{2}}$$

### Subjects

The inclusion criteria of the study were mental and physical fitness, being literate, and willingness to participate in the study. The subjects who missed more than two educational program sessions were excluded.

**Fig. 1:**
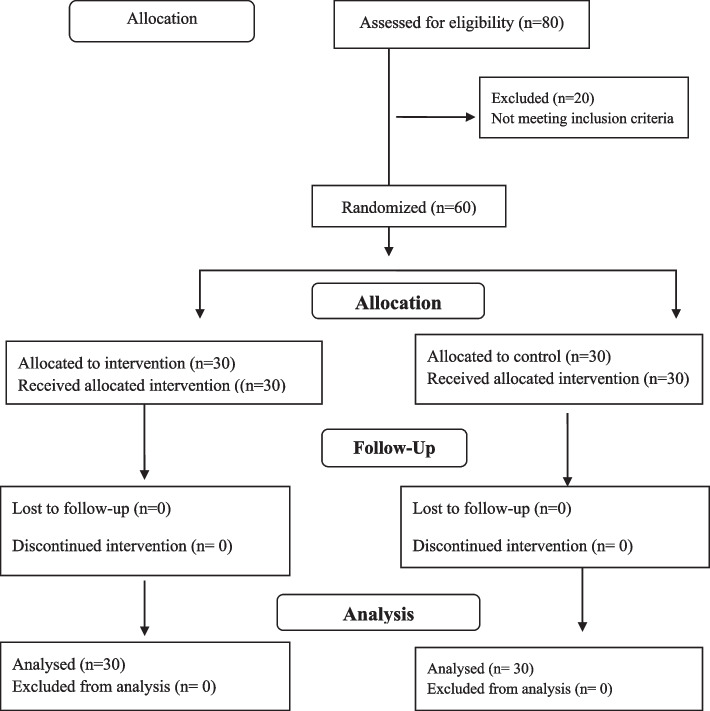
Flow diagram of the participant

Initially, 80 firefighters were selected via convenience sampling and invited to participate in the study. Subsequently, 20 of the firefighters who were not willing to participate in the study or did not meet the inclusion criteria were excluded. The remaining 60 firefighters were randomly allocated to two groups: workshop and simulation. To allocate firefighters to the workshop and simulation groups, 60 cards were used. The cards were marked A or B, with 30 cards marked A and 30 marked B. Firefighters were asked to draw one card from an envelope. Those who drew a card marked A were assigned to the workshop group, while those who drew a card marked B were assigned to the simulation group. Figure 1 displays a flow diagram of the participants throughout the study.

### Measurement

Data were collected using a demographics survey, a basic CPR knowledge questionnaire, and an observation checklist for CPR performance. The basic CPR knowledge questionnaire consisted of 15 multiple-choice questions. Each correct answer earned 1 point, while 0 was assigned to wrong answers or unanswered questions. Thus, the total score range was between 0 and 15. The observation checklist for CPR performance was comprised of 15 items. Each correct answer to the performance questions was given 1 point, and a point of 0 was assigned to incorrect performance or no performance. Thus, the total score range was between 0 and 15. The basic CPR knowledge questionnaire and the observation checklist for CPR performance were prepared according to the AHA guidelines 2020 [19].

The face and content validity of the basic CPR knowledge questionnaire as well as the observation checklist were tested and confirmed. In order to test content validity, the researchers measured the content validity ratio (CVR) and content validity index (CVI) of the scales. The necessity of the items was determined by a panel of experts who were asked to rate the items as ‘necessary’, ‘useful but not necessary’, and ‘not necessary’. The items whose values were greater than 0.49 were considered acceptable based on Lawshe table [20]. The CVR values of 15 items from the CPR knowledge questionnaire and the observation checklist for CPR performance were higher than 0.49. With regard to CVI, 15 experts were asked to evaluate the items in terms of relevance, clarity, and simplicity. Scores above 0.79 were considered acceptable [21]. The CVIs of 15 items from the CPR knowledge questionnaire and the observation checklist for CPR performance were found to be greater than 0.79.

The reliability of the questionnaire was measured using the test–retest method. Accordingly, 50 firefighters were asked to complete the questionnaire twice with a two-week interval. The intra-class correlation coefficient (ICC) across the 15 items was 0.79, which verified the internal consistency of this questionnaire [22]. The inter-observer reliability of the observation checklist equaled 0.87, which proved the checklist to be reliable.

The questionnaires, which were distributed by one of the co-researchers who was not aware of the allocation of the participants, were completed by the participants before, immediately after, and three months, post-intervention.

### Intervention

The participants who were educated via workshops attended four 45-min sessions. In these workshops, the educational content was presented through lectures and PowerPoint slides, based on AHA 2020 guidelines. Each workshop was followed by a Q & A session in which the firefighters’ questions were answered. The practical test was also carried out as an Objective Structured Clinical Examination (OSCE) on the simple standard training manikin at the University's clinical skills workshop with all firefighters evaluated based on a pre-prepared checklist.

As for the simulation-based intervention, the researchers used a smart CPR training manikin. Designed to imitate the anatomy of a human, the manikin was equipped with a monitor which displayed the progress of CPR. The monitor allowed the firefighters to correct their mistakes and learn to perform CPR correctly. All participants in the simulation group took turns in pairs and practiced the five steps involved in CPR individually according to the AHA model. Each of the firefighters was given the chance to perform a complete CPR on the simulator to achieve competence and be able to give CPR free of any errors.

At the University’s clinical skills workshop, a practical test was conducted on an advanced standard training manikin equipped with virtual reality (VR) glasses built by USA NASCO company in a simulated group. The manikin had markers embedded in it which allowed people to identify their errors during CPR and correct their behavior. Additionally, by connecting the mannequin to the monitor and designing various scenarios, firefighters became familiar with the main content of the BLS, and examiner can see as well as correct CPR function. Finally, the skills of all firefighters were evaluated based on a pre-prepared checklist. The simulation training was carried out in four 45-min sessions in a training room.

### Statistical analysis

The data were analyzed using IBM SPSS 26.0 statistical software. A descriptive analysis of the continuous variables was carried out, expressed as means and standard deviations. In order to evaluate the normality of each continuous variable, the Kolmogorov–Smirnov test was performed. The related variables were analyzed using the Repeated measures t-tests, Samples t-tests, and Chi-squared tests. Level of significance was set at *P* < 0.05.

### Ethical considerations

The present study was conducted in accordance with the principles of the revised Declaration of Helsinki, a statement of ethical principles that directs physicians and other participants in medical research involving human subjects. Prior to performing the interviews, all subjects were informed about the objectives of the study, the voluntary nature of their participation, the data collection methods, the reason of recording the interviews, the role of the interviewer and the participants, as well as confidentiality and anonymity of the information. Subsequently, they were asked to sign the informed consent form if they were willing to participate. The participants were notified that they are free to withdraw from the study at any time. Further, the study was approved by the Institutional Research Ethics Committee of Fasa University of Medical Sciences, Fasa, Iran (ethical code: IR.FUMS.REC. 1401.083).

## Results

In the present study, of the 80 firefighters selected, 20 firefighters did not meet the inclusion criteria. Thus, 60 firefighters were qualified to be evaluated. All participants were male since firefighting is an exclusively male domain in Iran. As for the participants’ demographic characteristic, their education, marital status, history of participation in workshops, age, and work experience were examined. The chi-squared test and independent t-test revealed that all characteristics of the experimental and control groups were the same, and there was no significant difference in these two groups (*P* < 0.05) (see Table 1). The results indicated that, in the pretest stage, there was not a statistically significant difference between the knowledge and skill mean scores of the two groups (0.988 and 0.927 respectively) and the groups were homogeneous. To lessen the impact of history of participation in workshops on the participants’ CPR knowledge and skill mean scores, the researchers applied the repeated measure test.

**Table 1:** Comparison of the firefighters’ demographic characteristics between the intervention and control groups

Variable		Workshop		Simulation		*P*-value
		Frequency	Percentage	Frequency	Percentage	
Education	High school	11	36.7	11	36.7	0.52
	Associate degree	3	10	7	23.3	
	Bachelor’s	15	50	11	36.7	
	Master’s or PhD	1	3.3	1	3.3	
Marital status	Married	18	60	16	53.3	0.78
	Single	12	40	14	46.7	
Prior participation in workshops	Yes	18	60	25	83.3	0.65
	No	12	40	5	16.7	
Age	Mean ± SD	33.37 ± 9.25		33.73 ± 8.21		0.87
Work experience	Mean ± SD	8.52 ± 7.53		8.57 ± 6.83		0.75

Statistical analyses of the impact of education via workshops revealed that the workshop group’s knowledge mean score rose from 7.67 ± 2.1 before the intervention to 12.10 ± 0.92 after the intervention (*P* < 0.001); their skill mean score rose from 8.17 ± 1.51 before the intervention to 12.07 ± 0.58 after the intervention (*P* < 0.001). As measured three months after the intervention, the workshop group’s knowledge and skill mean scores were found to have declined to 11.57 ± 0.68 and 11.70 ± 0.59 respectively, with the reduction being statistically significant (*P* < 0.001) (Table 2). The Friedman and Mann–Whitney test results indicated significant differences across the workshop group’s knowledge and skill mean scores as measured before, immediately after, and three months, post-intervention (*P* < 0.001).

**Table 2:** A comparison between the workshop and simulation groups’ knowledge and skill mean scores before, immediately after, and three months after the intervention

Group		Pretest	Posttest	Follow-up	*P*-value^*^
Knowledge	Workshop	7.67 ± 2.1	12.10 ± 0.92	11.57 ± 0.68	< 0.001
	Simulation	7.63 ± 1.62	14.03 ± 0.92	12.6 ± 0.81	< 0.001
	*P*-value^**^	0.988	< 0.001	< 0.001	-
Skill	Workshop	8.17 ± 1.51	12.07 ± 0.58	11.70 ± 0.59	< 0.001
	Simulation	8.33 ± 1.32	13.83 ± 0.53	12.57 ± 0.68	< 0.001
	*P*-value^**^	0.927	< 0.001	< 0.001	-

^*^Repeated measures

^**^Independent t-test

Regarding the simulation group, the participants’ knowledge mean score increased from 7.63 ± 1.62 before the intervention to 14.03 ± 0.92 after the intervention, and their skill mean score rose from 8.33 ± 1.32 pre-intervention to 13.83 ± 0.53 post-intervention (*P* < 0.001). As measured three months after the intervention, the simulation group’s knowledge and skill mean scores were found to have dropped to 12.60 ± 0.81 and 12.57 ± 0.68 respectively, and the reduction was statistically significant (*P* < 0.001). The Friedman and Mann–Whitney test results demonstrated significant differences across the simulation group’s knowledge and skill mean scores as measured before, immediately after, and three months following the intervention (*P* < 0.001) (Table 2).

Statistical analyses showed a significant difference between the effectiveness of the two approaches of workshops and simulation in enhancing the personnel’s CPR knowledge and skill (*P* < 0.001): the knowledge mean score of the participants who were trained via simulation was 1.93 points higher than that of the participants who were trained via workshops. Also, the skill mean score of the participants who were trained via simulation was 1.76 points higher than that of the participants who were trained via workshops (Table 2).

## Discussion

The present study was conducted to compare the effectiveness of a simulation-based training course to that of the traditional method of workshops in enhancing the BLS knowledge and skill of two groups of firefighters. The findings revealed a significant increase in the posttest scores of both groups as measured immediately after and three months after the interventions. However, simulation-based training was found to be considerably more effective than workshop training in enhancing the participants’ basic CPR knowledge and skill. This finding is consistent with the results of other studies reporting that, among the learning approaches commonly used to enhance CPR knowledge and skills, simulation training is the most effective [23]. It was similarly found that there is a direct correlation between patients’ survival rate and increase in BLS simulation training courses [24]. In addition, studies of the impact of simulation on learning, a significant increase has been reported in the learners’ knowledge and technical skills [25–27], ease of use, boosting self-confidence, converting skills to actual practice [25], and contributing to self-efficacy plus clinical judgment. It has also been established that learners prefer simulation experiences to traditional methods of learning [26].

In their study, Kim & Keun-Ja (2023) compared the effects of virtual reality medical simulation to flipped learning on firefighters’ competence in CPR. They found that the participants in the medi-VR group achieved higher posttest CPR knowledge and performance scores than the participants in the flipped learning group [28]. Research suggests that simulation on real scenes can improve firefighters’ cognitive capacity for critical analysis of problems and emergency care management, and despite their far shorter medical training compared to other EMS personnel, firefighters can benefit from simulated emergency care training at appropriate difficulty levels [29].

Regardless of the number of incidents which an individual may experience in the course of his/her professional life, no two events are ever identical and intervening factors such as personality types, mental and financial conditions, external interventions, and the hazards which should be overcome vary. Addressing this variety in incidents depends on technical-scientific preparation, which can be achieved through workshops and on-the-job training courses [30].

In addition, there are high expectations of firefighters and their competence in coping with high-risk and life-threatening situations. Nevertheless, these situations are uncommon in firefighters’ professional lives, stressing the need for keeping their preparation levels high through regular training and practice [29]. In other words, as the infrequency of CPR cases in their job restricts firefighters’ chances for on-the-job training, simulation training is a strategy which allows for purposeful selection and continuing practice without putting patients’ safety at risk [31]. Thus, firefighters’ training cannot rely entirely on traditional methods of transferring knowledge and there is an obvious need for hands-on learning [32]. Stimulation is a way to practicing and retaining skills which are rarely used. In the simulation of medical emergencies, responders are challenged to execute critical analyses of problems and make decisions in a complicated and rapidly changing environment. The available data are often restricted or contradictory, so simulation is a key part of personnel training in medical emergency contexts [29].

Previous studies of professionals other than firefighters, e.g. nurses and nursing students, have shown that the participants who were trained in advanced life support (ALS) via simulation achieved higher knowledge and performance scores than those who were educated via lecturing [33–35]. Also, studies have shown that BLS simulation programs are an effective way to teaching BLS skills to nursing students [36–38]. Similar results were reported by Tobase et al. (2017): an online BLS simulation-based course using instant feedback devices resulted in higher CPR scores and the course proved an effective method for learning key BLS skills [39]. Oermann et al. (2011) concluded the even brief CPR drills on manikins with corrective feedback can contribute to improving and maintaining one’s CPR skills [37]. Research also shows that high-tech simulation manikins for BLS training are effective learning tools which should be used more frequently [24]. According to another study, a simulation-based CPR learning program enhance clinical nurses’ CPR knowledge and performance as well as reduces their stress while performing CPR [40]. A study by Laco et al. (2022) found that simulation training with a high-fidelity manikin enhanced the BLS scores and teamwork perception of doctors, nurses, and medical technicians [41]. It has also been found that simulation training contributes to other medical skills, including inserting central venous catheter [42, 43], thoracentesis [44], and lumbar puncture (LP) skills [45].

These findings can be attributed to the fact that, in contrast to lecturing, simulation allows for more practice, more effective learning, and feedback from instructors, which results in positive cognitive outcomes [34]. Simulation is an active learning approach with positive effects on thinking skills and lifetime learning [46]. It also creates a safe and unthreatening environment where learners can improve their leadership, critical thinking, decision-making, problem-solving, and prioritization skills [47]. In a meta-analysis, simulation training for resuscitation performed by healthcare experts proved very effective, which was the result of the design features of “booster” practice, team/group dynamics, distraction, and systematic feedback [31]. In general, simulation training in CPR skills, regardless of target variables, learners’ levels, study design, or the responsibilities of the trainees, is an effective method and improves knowledge skills, process skills, product skills, time skills, and patient outcomes [31].

In contrast to the findings of the present study, a study by Castillo et al. (2023) found that, among the first-year students at a medical school, the outcomes of learning via virtual reality simulation were similar to those of the traditional method of face-to-face learning, and even with regard to defibrillation, traditional learning yielded better results. The researchers reported that virtual reality simulation had no impact on improving the learners’ BLS-AED knowledge. Further, manikin-based evaluations of the two groups’ skills showed that the difference between their scores was not significant [48].

In the present study, both methods of learning, i.e. simulation and workshop training, were found to be effective on enhancing the firefighters’ CPR knowledge and skill, which agrees with the belief that all forms of education can result in learning, though the depth and consistency of learning vary across different learning approaches [49]. One study compared the quality of CPR as performed by firefighters in a regular CPR drill to a simulated lifesaving event. The results were satisfactory in both groups, which can be attributed to the firefighters’ traditional training. Regular training in CPR helps firefighters preserve their skills over time [50]. Abelsson et al. (2019) found that six sessions of training per month improved ventilation volume, compression depth and rate, and recoil. The researchers concluded that visual feedback in the course of their training improved the firefighters’ CPR skills [51]. A study by Donizeti Silva et al. (2023) reported that there was a moderate correlation between the number of training courses taken by firefighters and a decline in their mental fatigue during two-minute CPR attempts. This finding indicates that better technical-scientific preparation results in experiencing less difficulty and fatigue during CPR. More training in BLS enables firefighters to perform CPR more effectively with a focus on ETCs and for longer periods before voluntary cessation of action due to fatigue [30]. In general, awareness and practical training are the principles of successful CPR [25].

In the present study, the results revealed a decline in the CPR knowledge and skill of both groups as assessed three months, post-intervention, which is consistent with the findings of other studies, confirming that CPR knowledge and adequacy of skills are not permanent [47], and lasting retention of BLS knowledge and skill over time is unlikely [24, 52]. This contributes to low survival rates following cardiac arrest [52]. As with the present study, a study by Castillo et al. (2023) found an increase in the participants’ CPR skills after traditional training and simulation training, but they declined over time [48]. Similarly, Toubasi et al. (2015) reported that retention of BLS skills after training diminishes in the course of time and that corrective measures, including compulsory renewal of BLS certificates, are essential [46]. In another study, 10 weeks after training in CPR, the participants’ CPR knowledge and skills were found to have declined; however, as with the present study, the learners’ scores were still higher than their pretest scores, which demonstrated positive retention of cognitive knowledge of CPR and psychomotor skills [53]. In addition, literature shows that, compared to theoretical knowledge of CPR, CPR practical skills decline more rapidly [24, 54], and that advanced cardiac life support (ACLS) skills wane faster than BLS skills [54]. Other studies report that the adequacy of skills following CPR training is highly variable and that retention of skills declines after a certain period [36, 47, 53, 55, 56], and CPR skills are weakened after three to six months [41, 57, 58]. Considering the decline in levels of knowledge and skill, CPR training courses should be repeated over time [25, 49, 59, 60]. There is a consensus about the necessity of regular training in CPR, and frequent short-term training courses are highly recommended [39–50]. Greater exposure to content correlates with better compliance with the principles of life support [39]. Some researchers suggest that CPR training should be repeated every three to six months to avoid decline in first responders’ knowledge and skills [24–47]. In conclusion, low-quality CPR is a preventable event. Use of technological advances in learning and employment of effective educational resources, e.g. simulators, providing feedback and promoting teamwork during simulation and repetition of training courses can contribute to the CPR knowledge and skills of first responders, including firefighters, especially in out-of-hospital environments.

### Limitations

In the present, the long-term effects of the educational intervention after three months were not investigated. More studies are required to verify the durability of the outcomes of these interventions. However, it is evident that training courses should be repeated periodically to guarantee the retention of CPR knowledge and skills. Another limitation of the study has been lack of literature on the effects of simulation-based CPR interventions on professional firefighters.

### Strengths

Only a few studies have addressed and compared the impacts of simulation training as well as training via workshops on firefighters’ CPR knowledge and skills. In view of the significant role of professional firefighters in performing CPR in out-of-hospital environments and the correlation between the quality of their performance and rate of deaths caused by cardiac arrest, the findings of the present study can prove very helpful. It is suggested that future studies address the optimal number of and intervals between BLS training sessions, as well as additional strategies for improving as well as maintaining CPR knowledge and skills in firefighters and other emergency workers.

## Conclusion

The findings of the present study revealed that, though both methods of simulation training and workshops were effective, simulation-based training in BLS was certainly a more effective method for promoting firefighters’ BLS knowledge and skills as it allowed the participants to practice the different steps of BLS in simulated CPR exercises and receive feedback. Thus, as an effective approach to clinical learning, simulation can provide invaluable experiences to firefighters as well as their instructors and managers, enhance firefighters’ knowledge and psychomotor skills, improve their critical thinking and decision making skills, and ultimately enhance patients’ chances of survival, especially outside hospitals. Considering the decline in firefighters’ CPR knowledge and skills over time and the need for keeping firefighters abreast of changes in knowledge and international standard guidelines, repetition of simulation-based training and even traditional methods of training in CPR for this population, who are among the first responders engaging in out-of-hospital resuscitation in emergencies, is strongly recommended.

## Data Availability

The datasets generated and/or analysed during the current study are not publicly available due to the necessity to ensure participant confidentiality policies and laws of the country but are available from the corresponding author on reasonable request.

## Abbreviations

CPR: Cardiopulmonary resuscitation
BLS: Basic life support
ACLS: Advanced cardiac life support
